# Urolithin A enhances mitochondrial biogenesis-related markers and maximal respiratory capacity during C2C12 differentiation

**DOI:** 10.3389/fcell.2026.1854844

**Published:** 2026-06-30

**Authors:** Ronald Vargas-Foitzick, Diego Irribarra-Tapia, Mayalen Valero-Breton, Elisa Balboa

**Affiliations:** 1 Exercise Science Laboratory, School of Kinesiology, Faculty of Medicine, Universidad Finis Terrae, Santiago, Chile; 2 Escuela de Ciencias de la Salud, Departamento de Kinesiología, Facultad de Medicina, Pontificia Universidad Católica de Chile, Santiago, Chile; 3 Center for Biomedical Research, School of Medicine, Faculty of Medicine, Universidad Finis Terrae, Santiago, Chile; 4 Exercise and Rehabilitation Sciences Institute, Faculty of Rehabilitation Sciences, School of Physical Therapy, Universidad Andres Bello, Santiago, Chile; 5 Institute of Health and Sport Sciences, Faculty of Health Science, Universidad Francisco de Vitoria, Madrid, Spain

**Keywords:** C2C12 myotubes, maximal respiration, myogenic differentiation, oxphos, urolithin A

## Abstract

**Introduction:**

Skeletal muscle differentiation in the C2C12 myoblast model requires extensive mitochondrial remodeling to meet rising bioenergetic demands through coordinated changes in biogenesis, dynamics, and respiratory adaptation. Urolithin A (UA), a gut microbiota-derived metabolite of ellagitannins, improves mitochondrial health, but its role in late-stage myogenic differentiation remains unclear.

**Methods:**

C2C12 myotubes were treated with UA (2 μM) for 72 h during late-stage differentiation (days 3–6). Mitochondrial signaling, respiratory capacity, myogenic morphology, and ultrastructure were assessed by Western blot, high-resolution respirometry, hematoxylin–eosin staining, and transmission electron microscopy.

**Results:**

UA was non-cytotoxic and increased AMPKα phosphorylation and PGC-1α expression, whereas TOM20, MFN2, and OPA1 were unchanged. Mitophagy/autophagy-related markers (p-ULK1, p62, BNIP3L/NIX, LC3-II/I) were not altered, indicating no detectable changes in steady-state autophagy under the conditions tested. UA selectively increased OXPHOS Complex I and II abundance and enhanced maximal uncoupled respiration, and was associated with increased myotube diameter and myogenic marker abundance. No overt ultrastructural differences were observed by electron microscopy.

**Discussion:**

These findings suggest that UA promotes mitochondrial functional adaptation during myogenic differentiation, with accompanying changes in myogenic phenotype, without clear evidence of altered steady-state mitophagy/autophagy markers or mitochondrial morphology.

## 1 Introduction

Skeletal muscle differentiation is an energy-demanding process requiring extensive remodeling of cellular structure, metabolism, and organelle function during which myoblasts differentiate and fuse to form multinucleated myotubes. This transition is accompanied by a marked increase in mitochondrial content and oxidative capacity, supporting a metabolic shift from glycolysis toward oxidative phosphorylation (OXPHOS) as the primary source of ATP (Sin et al., 2016; Remels et al., 2010; Wang et al., 2016), depending on coordinated mitochondrial quality control, including mitophagy for damaged organelle clearance, biogenesis, and network reorganization. Given the high energetic demand and mitochondrial dependency of skeletal muscle differentiation, strategies aimed at enhancing mitochondrial function and metabolic efficiency may represent a promising approach to improve myogenic progression and muscle regeneration. This concept is particularly relevant in physiological and pathological contexts characterized by increased muscle remodeling demands, such as recovery from muscle injury, aging-associated muscle decline, or high-performance athletic adaptation, where accelerated or enhanced differentiation could contribute to improved functional recovery and muscle performance (Serova et al., 2024). However, despite the recognized importance of mitochondrial remodeling during myogenesis, the way in which bioactive compounds modulate this process during late-stage differentiation remains insufficiently defined.

The C2C12 myoblast model is widely used to study mammalian myogenesis due to its robust differentiation and conserved mitochondrial dynamics (Sin et al., 2016; Remels et al., 2010). During C2C12 differentiation, mitophagy predominates during early stage (days 1–3), facilitating clearance of dysfunctional mitochondria, followed by increased biogenesis and network maturation at intermediate/late stages (days 3–6) (Sin et al., 2016; Remels et al., 2010; Wang et al., 2016). This temporal shift makes the late differentiation window particularly relevant for investigating how compounds influence the balance between organelle turnover and metabolic maturation. In this context, BNIP3L/NIX and PINK1-Parkin pathways mediate mitophagy to selectively remove damaged mitochondria, while mitofusin-2 (MFN2) and OPA1 regulate fusion and cristae organization (Tezze et al., 2017; Sebastián et al., 2016). Disruption of these processes impairs myogenic progression and myotube function (Tezze et al., 2017; Sebastián et al., 2016; Rochard et al., 2000), underscoring the essential role of mitochondrial remodeling in functional myotube formation. This temporal window is therefore especially useful for determining whether candidate compounds act on mitochondrial turnover, metabolic adaptation, or both.

Urolithins, gut microbiota-derived metabolites of ellagitannins from pomegranates and walnuts, are converted into several metabolites, including urolithin A, B, C, and D (Gonzalez-Jamett et al., 2022). Urolithin A (UA) is the predominant circulating metabolite detected in human plasma following consumption of ellagitannin-rich foods (Tomás-Barberán et al., 2014), with reported concentrations ranging from 0.024 to 35 μM depending on microbiota composition and polyphenol intake (Singh et al., 2022). UA is associated with improved mitochondrial function and cellular homeostasis (Tang et al., 2017; Andreux et al., 2019; Ryu et al., 2016). Preclinical studies in *Caenorhabditis elegans* demonstrated that UA induces mitophagy, preserves mitochondrial function, and improves mobility and lifespan (Ryu et al., 2016). These effects translate to rodent models, where UA supplementation enhances exercise capacity in both age-related muscle decline and young animals (Ryu et al., 2016). In randomized, placebo-controlled clinical trials, UA administration in middle-aged and older adults is safe and associated with improvements in muscle strength, endurance, and physical performance (Singh et al., 2022).

Despite growing evidence supporting UA’s effects on mitochondrial health in mature muscle and systemic models, a critical translational gap remains: it is unknown whether UA can modulate mitochondrial adaptation during the active phase of myogenic differentiation. Current translational models relying on fully differentiated or mature muscle systems do not capture this dynamic remodeling phase, which limits their applicability to regenerative contexts. The C2C12 late-stage differentiation model (days 3–6) provides a tractable *in vitro* system to isolate and characterize UA’s effects during this specific biological window, establishing a mechanistic foundation prior to more complex *in vivo* investigation.

Therefore, this study investigated whether 2 μM UA, selected as a non-cytotoxic concentration within the physiologically relevant plasma range reported after oral UA supplementation in humans, modulates mitochondrial signaling, respiratory capacity, myogenic morphology, and ultrastructure during late-stage C2C12 differentiation (days 3–6).

## 2 Materials and methods

### 2.1 Cell culture

The murine C2C12 cell line (ATCC) was used. C2C12 myoblasts were seeded at a density of 1 × 10^5^ cells per well in 6-well plates. Cells were cultured in DMEM (Life Technologies) supplemented with 10% fetal bovine serum (GIBCO), 2% penicillin/streptomycin (GIBCO), 1% non-essential amino acids (NEEA; GIBCO), and 0.1% amphotericin B (GIBCO) in a humidified atmosphere with 5% CO_2_ at 37 °C. Upon reaching ∼70% confluence, the proliferation medium was replaced with a differentiation medium containing 2% horse serum (GIBCO), 2% penicillin/streptomycin, 1% NEEA, and 0.1% amphotericin B. The differentiation medium was refreshed every day.

On day 3 of differentiation, cells were treated with Urolithin A (2 μM; BOC Sciences, United States), prepared in DMSO (final concentration 0.1% v/v). DMSO was used as vehicle control during differentiation. UA treatment was initiated on day 3 and maintained for 72 h, with medium replacement every 24 h.

### 2.2 Cell viability

Cell viability was determined using the MTT assay (Invitrogen) according to the manufacturer’s instructions. C2C12 myotubes were treated on day 3 of differentiation with increasing concentrations of Urolithin A (0, 0.2, 2, 5, 10, and 20 μM) for 72 h.

At the end of the treatment period, MTT solution (1.2 mM) was added to the cells. After 4 h of incubation at 37 °C, the resulting formazan crystals were dissolved in 100 μL of 0.1% SDS-HCl solution and incubated for 18 h in a humidified chamber. Absorbance was measured at 570 nm. Cell viability was expressed as a percentage relative to the control condition, which was set at 100%.

### 2.3 Histological assessment

After differentiation, cells were washed twice with PBS and fixed with 4% paraformaldehyde (PFA) for 5 min at room temperature. Fixed cells were then stained with hematoxylin for 3–5 min, washed twice with running water, and counterstained with eosin for 1 min. After eosin staining, samples were washed three times with distilled water, air-dried, and imaged for subsequent analysis.

Hematoxylin and eosin (H&E)-stained preparations were used to assess myotube morphology and quantify differentiation-related structural features. The myogenic index was calculated as the percentage of nuclei contained within myotubes relative to the total number of nuclei, using the formula (number of nuclei in myotube/total number of nuclei) x 100. Myotube diameter was quantified using ImageJ software. For each biological replicate, 10 myotubes per field were analyzed across seven randomly selected fields, and the average diameter of each myotube was obtained from measurements taken at five different points along the cell length (Ono and Sakamoto, 2017). Quantitative analysis of H&E-stained images was performed by an evaluator blinded to the experimental conditions to reduce observer bias.

### 2.4 Protein extraction

At the end of the differentiation period, myotubes were rinsed once with PBS and harvested in ice-cold lysis buffer containing 20 mM Tris (pH 7.0), 270 mM sucrose, 5 mM EGTA, 1 mM EDTA, 1% Triton X-100, 1 mM sodium orthovanadate, 50 mM sodium β-glycerophosphate, 5 mM sodium pyrophosphate, 50 mM sodium fluoride, 1 mM DTT (dithiothreitol), and a protease inhibitor cocktail containing 1 mM EDTA (Roche Applied Science, Belgium).

Homogenates were centrifuged at 12,500 rpm for 10 min at 4 °C, and the resulting supernatants were collected and stored at −80 °C. Protein concentration was determined using the DC Protein Assay Kit (Bio-Rad).

### 2.5 Western blot analyses

Cell lysates (18 μg of protein) were mixed with Laemmli sample buffer and heated for 5 min at 95 °C prior to loading onto 12% SDS-PAGE gels. Proteins were separated by electrophoresis at 120 V for 1.5 h and subsequently transferred onto PVDF membranes at 80 V for 1.5 h for Western blot analysis.

Membranes were blocked for 60 min in Tris-buffered saline containing 0.1% Tween-20 (TBST) and 5% non-fat milk. Membranes were then incubated overnight at 4 °C with the following primary antibodies: rabbit anti-phospho-AMPKα (Thr172) (1:1,000, 2603S, Cell Signaling Technology), mouse anti-PGC-1α (1:500, SC-517380; Santa Cruz, Dallas, TX, United States), rabbit anti-TOM20 (1:1,000, 42406S, Cell Signaling Technology, Danvers, MA, United States), rabbit anti-phospho-ULK1 (1:1,000, 14202S, Cell Signaling Technology), rabbit anti-BNIP3L (1:1,000, 12396S, Cell Signaling Technology), LC3 (1:1,000, 4,108, Cell Signaling Technology, Danvers, MA, United States), p62 (1:2000, H00008878-M01, Abnova), mouse anti-Total OXPHOS cocktail (1:1,000, ab110413, Abcam), mouse anti-MFN2 (1:1,000, ab56889, Abcam), mouse anti-optic atrophy protein 1 (OPA1) (612606, BD Biosciences), and mouse anti-MYH (1:500, SC-47724; Santa Cruz, Dallas, TX, United States), mouse anti-MYH (1:500, SC-376157; Santa Cruz, Dallas, TX, United States), mouse anti-MyoD (1:500, SC-377460; Santa Cruz, Dallas, TX, United States), mouse anti-myogenin (1:500, SC-47724; Santa Cruz, Dallas, TX, United States), mouse anti-MRF4 (1:500, SC-47724; Santa Cruz, Dallas, TX, United States).

Membranes were then incubated with the appropriate secondary antibodies: goat anti-mouse IgG-HRP (1:10,000; 31430, Invitrogen, United States) or goat anti-rabbit IgG-HRP (1:10,000; 7074P2, Cell Signaling Technology, Danvers, MA, United States). After three additional washes, protein bands were detected using an enhanced chemiluminescence (ECL) kit (Bio-Rad). Membranes were imaged using a ChemiDoc MP™ system (Bio-Rad), and band intensities were quantified using ImageJ software (NIH, Bethesda, MD, United States).

### 2.6 Oxygen consumption

Oxygen consumption rates (OCR) were measured using an Oxygraph-2k (OROBOROS Instruments, Innsbruck, Austria) in intact C2C12 myotubes. On day 6 of differentiation (72 h post-Urolithin A treatment), myotubes from each condition were trypsinized, and resuspended in DMEM (Life Technologies). Respiration was recorded sequentially under the following conditions: basal respiration (routine state); oligomycin (2 μM) to inhibit ATP synthase (leak respiration); FCCP (0.5 μM, titrated) to stimulate maximal uncoupled respiration (electron transport system capacity); and rotenone (0.5 μM) plus antimycin A (2.5 μM) to inhibit complexes I and III (residual non-mitochondrial respiration).

Data were acquired using DatLab 7 software and analyzed to determine basal respiration, oligomycin-sensitive respiration (ATP-linked), maximal uncoupled respiration (FCCP), and spare respiratory capacity. OCR values were normalized to cell number and expressed as pmol/(s·million cells). Experiments were performed in duplicate chambers per biological replicate (n = 5 per condition).

### 2.7 Transmission electron microscopy

Myotubes were treated in the absence or presence of Urolithin A (UA). After 72 h of treatment, myotubes were dissociated using trypsin (Thermo Fisher Scientific), centrifuged, and the resulting pellet was fixed in 2% glutaraldehyde. The fixed pellet was sectioned into fiber bundles and analyzed by transmission electron microscopy (TEM; Talos F200C G2, Microscopy Facility, Pontificia Universidad Católica de Chile) (Castro-Sepulveda et al., 2023).

For each independent experiment, mitochondria from five myotubes were analyzed. Mitochondrial density was calculated as the percentage of mitochondrial area relative to total cellular area, and mitochondrial size was expressed in nm^2^. The width-to-length ratio was calculated to assess mitochondrial circularity. All quantifications were performed in a blind manner by an expert at ×13,500 magnification.

### 2.8 Statistical analysis

Statistical significance was assessed using non-parametric tests for two-group comparisons (Mann–Whitney U test) and for three or more groups (Kruskal–Wallis test followed by Dunn’s multiple comparisons test). Mitochondrial respiration data were analyzed using two-way ANOVA (condition × substrate), followed by Sidak’s multiple comparisons test.

Data are presented as mean ± standard deviation (SD) for viability assays and as median with interquartile range (IQR) for protein expression, respirometry, and morphological analyses. The number of biological replicates (n) is indicated in each figure legend. All statistical analyses were performed using GraphPad Prism 8.0 (GraphPad Software, San Diego, CA, United States). A p-value <0.05 was considered statistically significant.

## 3 Results

### 3.1 C2C12 differentiation and effect of urolithin A on cell viability

**FIGURE 1:**
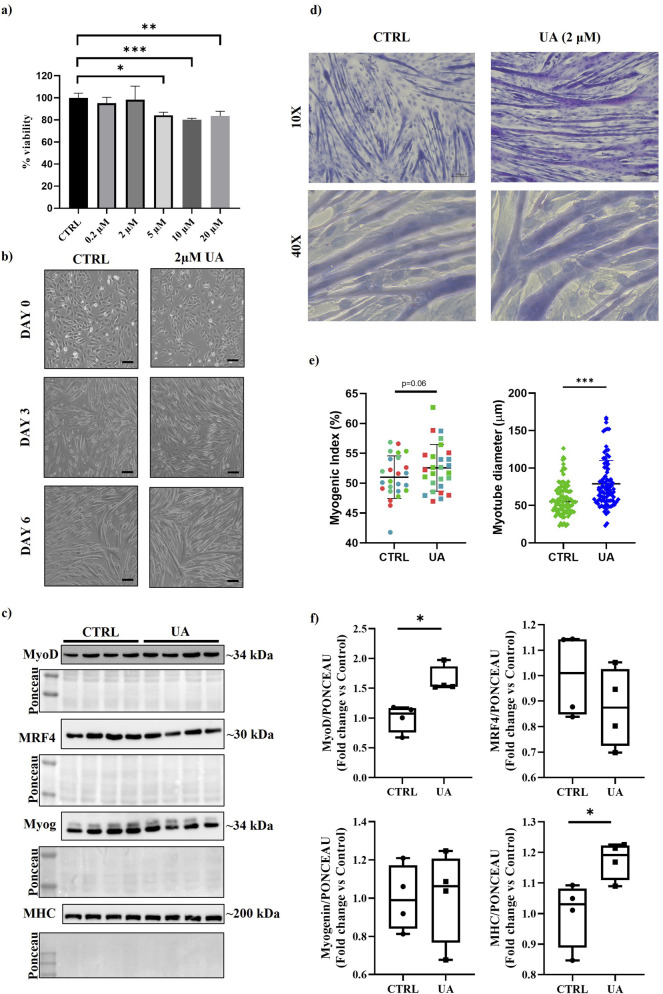
C2C12 differentiation and effects of Urolithin A on cell viability. **(a)** MTT viability assay. C2C12 cells were exposed to increasing concentrations of Urolithin A (UA; 0.2, 2, 5, 10, and 20 μM) for 72 h starting on day 3 of differentiation. Cell viability is expressed as a percentage relative to CTRL **(b)** Phase-contrast microscopy (10×, inverted) showing myoblast-to-myotube progression at day 0 (myoblasts), day 3 (myocytes), and day 6 (myotubes) (CTRL vs. 2 μM Urolithin A [UA] treatment during days 3–6). Scale bar: 100 μm. **(c)** Representative immunoblots of myogenic markers MyoD, MRF4, Myog, and MHC in CTRL and UA-treated cells. **(d)** Representative H&E-stained images of CTRL and UA (2 μM)-treated myotubes at ×10 and ×40 magnification. **(e)** Quantification of myogenic index and myotube diameter in CTRL and UA-treated cells. **(f)** Quantification of MyoD, MRF4, Myog, and MHC abundance normalized to Ponceau, shown as fold change relative to CTRL. Data are presented as mean ± SD (n = 6; Kruskal–Wallis test). ***p < 0.001, **p < 0.01, *p < 0.05 vs. CTRL.

To validate C2C12 myogenic differentiation and establish a non-cytotoxic UA concentration during days 3–6, cell viability was first assessed by MTT assay relative to CTRL (0.1% DMSO) (Figure 1a). UA at 0.2 and 2 μM did not affect viability, whereas concentrations ≥5 μM reduced viability by approximately 15% after 72 h. Accordingly, 2 μM UA was selected for subsequent experiments, and representative images were acquired to assess myotube morphology (Figure 1b).

To further characterize the biological effect of the selected UA concentration, we evaluated myogenic markers and myotube morphology during C2C12 differentiation (Figure 1c). UA increased MyoD and myosin heavy chain (MYH) abundance (Figure 1f) and was associated with a significant increase in myotube diameter (Figure 1e), whereas MRF4 and Myog were not significantly modified and the myogenic index showed no clear change (Figure 1e). Overall, these findings suggest that UA may selectively influence late myogenic features rather than broadly enhancing the differentiation program. We next investigated whether these changes were accompanied by alterations in mitochondrial remodeling and respiratory function in differentiated C2C12 cells.

### 3.2 Urolithin A does not alter mitophagy or autophagy-related proteins in C2C12 myotubes after 72 h of treatment (days 3–6 of differentiation)

**FIGURE 2:**
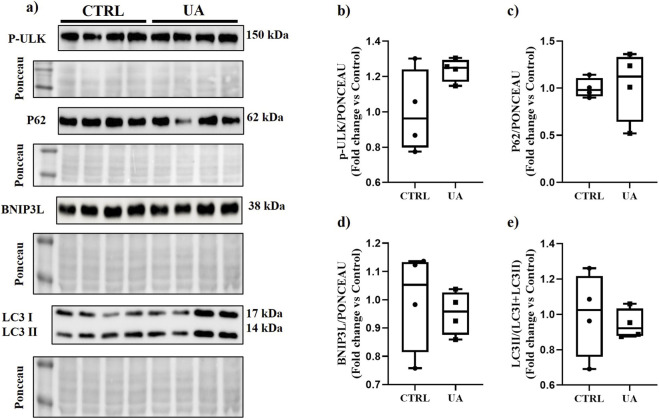
Urolithin A does not alter mitophagy/autophagy-related proteins in C2C12 myotubes. C2C12 myotubes were treated with 2 μM Urolithin A (UA) for 72 h starting on day 3 of differentiation (days 3–6). **(a)** Representative immunoblots and Ponceau staining. Quantification of Western blot band intensities are expressed as fold change relative to CTRL for p-ULK1 **(b)**, p62 **(c)**, BNIP3L/NIX **(d)**, and the LC3-II/LC3-I ratio **(e)**. Data are presented as median with interquartile range (Mann–Whitney test; *p < 0.05 vs. CTRL; n = 4).

To determine whether Urolithin A (UA) modulates mitophagy pathways during C2C12 differentiation, cells were treated with 2 μM UA for 72 h, and protein levels of key regulators of autophagy initiation and mitochondrial quality control were subsequently quantified by Western blot (Figure 2). Protein levels were normalized to total protein loading using Ponceau staining. To assess the autophagy machinery, phosphorylated ULK1 (p-ULK1), a key regulator of autophagy initiation, was analyzed. p-ULK1 levels did not differ between CTRL and UA-treated cells (1.00 ± 0.233 vs. 1.23 ± 0.06 AUC; p = 0.1) (Figure 2b), indicating that UA did not significantly affect ULK1 activation during days 3–6 of differentiation. Consistent with this observation, protein levels of p62, a selective autophagy receptor and cargo adaptor that binds ubiquitinated substrates for lysosomal degradation, were not significantly altered by UA treatment (1.00 ± 0.10 vs. 1.03 ± 0.37 AUC; p = 0.24) (Figure 2c), suggesting no major changes in autophagic steady state under these conditions. Similarly, the mitophagy-related protein BNIP3L/NIX showed comparable expression between groups (1.00 ± 0.17 vs. 0.95 ± 0.07 AUC; p = 0.34) (Figure 2d), indicating that receptor-mediated mitophagy pathways were not activated by UA at this stage of differentiation. Finally, the LC3-II/LC3-I ratio, an indicator of autophagosome formation, was not significantly altered (1.00 ± 0.23 vs. 0.94 ± 0.08 AUC; p = 0.24) (Figure 2e), further supporting the absence of major changes in autophagic steady state. Together, these results indicate that 3 days of UA exposure during late-stage C2C12 differentiation does not significantly modify the levels of key proteins involved in mitophagy or autophagy initiation, suggesting that UA-induced mitochondrial adaptations occur without detectable changes in steady-state markers of mitophagy/autophagy under the conditions tested.

### 3.3 Urolithin A upregulates mitochondrial biogenesis-related markers and fusion protein expression in differentiated C2C12 myotubes

**FIGURE 3:**
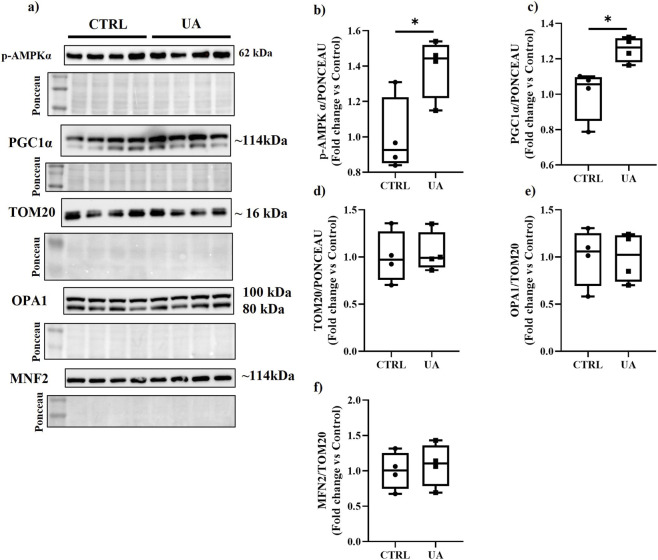
Urolithin A enhances mitochondrial biogenesis-related markers and fusion in differentiated C2C12 myotubes. C2C12 myotubes were treated with 2 μM Urolithin A (UA) for 72 h starting on day 3 of differentiation (days 3–6). **(a)** Representative immunoblots and Ponceau staining. Quantification of Western blot band intensities is expressed as fold change relative to CTRL for p-AMPKα **(b)**, PGC-1α **(c)**, TOM20 **(d)**, OPA1 **(e)**, and MFN2 **(f)**. Data are presented as median with interquartile range (Mann–Whitney test; *p < 0.05 vs. CTRL; n = 4).

To assess whether Urolithin A (UA) modulates mitochondrial content and dynamics, protein levels of key regulators of mitochondrial biogenesis and membrane remodeling were analyzed by Western blot. Representative immunoblots are shown in Figure 3a, and the corresponding quantification is presented in Figures 3b-f. Protein levels were normalized to total protein loading using Ponceau staining.

As a positive control of the reported effects of UA (Ryu et al., 2016), phosphorylated AMPKα (p-AMPKα) levels were significantly increased in UA-treated cells compared with CTRL (1.00 ± 0.21 vs. 1.39 ± 0.17 AUC; p = 0.02) (Figure 3b). Consistent with AMPKα activation, PGC-1α protein levels were also higher in UA-treated cells than in CTRL (1.00 ± 0.14 vs. 1.25 ± 0.07 AUC; p = 0.01) (Figure 3c). In contrast, TOM20 levels were unchanged in the UA group (1.00 ± 0.27 vs. 1.05 ± 0.21 AUC; p = 0.5) (Figure 3d). When normalized to TOM20, OPA1 protein expression remained comparable between groups (1.00 ± 0.30 vs. 0.99 ± 0.26 AUC; p = 0.5), indicating that OPA1 was not affected by UA at this stage of differentiation (Figure 3e). Similarly, MFN2 levels normalized to TOM20 were not significantly different between groups (1.00 ± 0.26 vs. 1.08 ± 0.30 AUC; p = 0.24) (Figure 3f).

### 3.4 Urolithin A upregulates OXPHOS Complex II and maximal respiratory capacity in differentiated C2C12 myotubes

**FIGURE 4:**
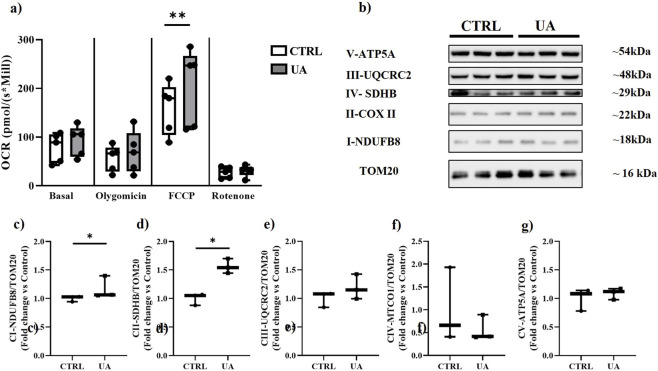
Urolithin A enhances mitochondrial respiratory function in differentiated C2C12 myotubes treated with 2 μM UA (days 3–6). **(a)** Mitochondrial oxygen consumption rates (OCR) measured by high-resolution respirometry (Oroboros O2k) under basal conditions, followed by sequential addition of oligomycin, FCCP, and rotenone/antimycin A, normalized to cell number and expressed as pmol/(s*Mill). **(b)** Representative immunoblots of OXPHOS complexes (I–V), with corresponding Ponceau S staining used as loading control. Quantitative analysis of Western blot band intensities expressed as fold change relative to CTRL for Complexes I **(c)**, II **(d)**, III **(e)**, IV **(f)**, and V **(g)**. Data are presented as median and interquartile range. Statistical significance was determined using two-way ANOVA followed by Sidak’s multiple comparisons test for respirometry evaluation (n = 5) and Mann–Whitney test for WB analysis (n = 4); *p < 0.05 vs. CTRL.

To determine whether Urolithin A (UA) modulates mitochondrial respiratory capacity in late-stage C2C12 myotubes, High-resolution respirometry data are shown in Figure 4a, and representative immunoblots of OXPHOS complexes in Figure 4b. Quantification of complexes I-V is presented in Figures 4c-g, including Complex V in Figure 4g. Respirometry data were analyzed using two-way ANOVA, with condition (CTRL vs. UA) and substrate as factors. A significant main effect of condition was observed (p = 0.0033), indicating that UA treatment increased mitochondrial respiration compared to CTRL. A significant main effect of substrate was also detected (p = 0.0001), confirming appropriate responses to the sequential substrate–inhibitor protocol. No significant interaction between condition and substrate was found (p = 0.0745), suggesting that UA enhanced mitochondrial respiratory capacity in a global manner rather than selectively affecting specific respiratory states. Sidak’s multiple comparisons test indicated that UA treatment did not significantly alter basal respiration, oligomycin-sensitive respiration, or non-mitochondrial respiration compared to CTRL. In contrast, UA significantly increased maximal respiration (FCCP) (158.6 ± 53.13 vs. 203.7 ± 79.07 pmol/(s·million cells); p = 0.003) (Figure 4a), indicating enhanced maximal mitochondrial respiratory capacity.

In line with the OCR results, UA treatment increased the abundance of selected OXPHOS complexes relative to CTRL. Specifically, UA significantly increased the protein levels of complex I (1.00 ± 0.04 vs. 1.17 ± 0.19 AUC; p = 0.05) (Figure 4c) and Complex II (1.00 ± 0.10 vs. 1.56 ± 0.12 AUC; p = 0.05) (Figure 4d). However, no significant changes were observed in Complex III (1.00 ± 0.13 vs. 1.18 ± 0.21 AUC; p = 0.2) (Figure 4e), Complex IV (1.00 ± 0.81 vs. 0.56 ± 0.27 AUC; p = 0.35) (Figure 4f), or Complex V (1.00 ± 0.19 vs. 1.08 ± 0.09 AUC; p = 0.35) (Figure 4). Together, these results suggest that UA enhances mitochondrial respiratory capacity during myogenic differentiation.

### 3.5 Urolithin A does not alter mitochondrial morphology in differentiated C2C12 myotubes

**FIGURE 5:**
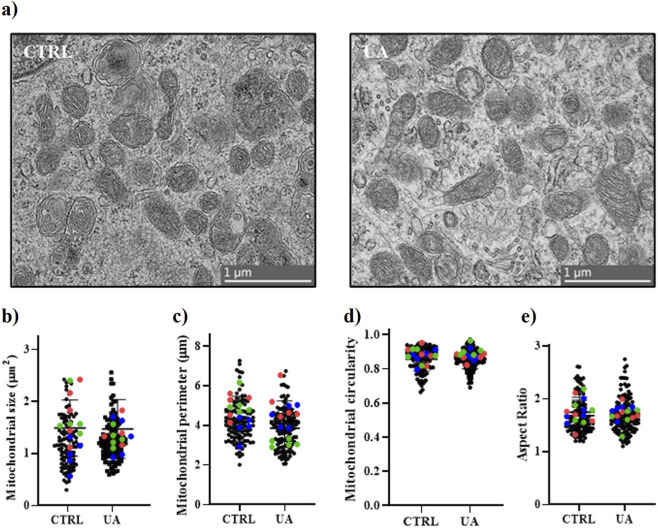
Urolithin A does not induce ultrastructural changes in mitochondrial morphology in differentiated C2C12 myotubes. C2C12 myotubes were treated with 2 μM Urolithin A (UA) for 72 h starting on day 3 of differentiation (days 3–6). **(a)** Representative transmission electron microscopy (TEM) images of CTRL and UA-treated samples (scale bar: 1 μm). **(b)** Quantitative analysis of mitochondrial area, **(c)** perimeter, **(d)** circularity, and **(e)** aspect ratio. Data are presented as median with interquartile range (Mann–Whitney U test). Quantification was performed from n = 5 myotubes per condition, with ≥50 mitochondria analyzed per group. Black dots represent individual mitochondrial measurements, and colored dots represent the mean value for each condition.

To examine whether Urolithin A (UA) alters mitochondrial morphology during the late stage of myogenic differentiation, mitochondrial area, perimeter, circularity, and aspect ratio (AR) were assessed by transmission electron microscopy (TEM) and quantified from five myotubes per group. Representative electron micrographs are shown in Figure 5a, and the corresponding quantitative analyses are presented in Figures 5b-e. Although UA induced changes in mitochondrial protein expression and function, TEM analysis revealed no significant differences in mitochondrial morphology between CTRL and UA-treated cells. Specifically, mitochondrial area (1.20 ± 0.46 vs. 1.27 ± 0.39 μm^2^; p = 0.163; Figure 5b), perimeter (4.22 ± 1.03 vs. 4.07 ± 0.91 μm; p = 0.213; Figure 5c), circularity (0.85 ± 0.06 vs. 0.85 ± 0.05; p = 0.33; Figure 5d), and aspect ratio (1.68 ± 0.34 vs. 1.69 ± 0.33; p = 0.41; Figure 5e) were comparable between groups.

## 4 Discussion

The principal finding of this study is that Urolithin A (UA) modulates mitochondrial biogenesis-related markers and maximal respiratory capacity in differentiated C2C12 myotubes (days 3–6), a phase characterized by myotube maturation and predominant mitochondrial biogenesis (Sin et al., 2016; Remels et al., 2010), without altering steady-state levels of mitophagy/autophagy markers or mitochondrial ultrastructure. This pattern indicates that UA amplifies the mitochondrial remodeling that naturally accompanies myogenic differentiation, resulting in myotubes with a higher oxidative reserve. However, the consequences for long-term contractile performance and stress resistance remain to be determined.

In the present study, 2 μM UA was selected based on its lack of cytotoxic effects in C2C12 myotubes and its alignment with plasma concentrations reported after oral UA supplementation in humans (Singh et al., 2022; Andreux et al., 2019). While 2 μM UA had no effect on cell viability, higher concentrations applied for 72 h significantly reduced survival, suggesting that excessive exposure is detrimental during late-stage C2C12 differentiation (days 3–6). This is consistent with reports showing that high UA concentrations can reduce viability in human muscle *in vitro* models (Zhao et al., 2023), and may trigger pro-apoptotic signaling (El-Wetidy et al., 2021). Importantly, our H&E analysis showed that 2 μM UA increased myotube diameter, which is consistent with an effect on myotube maturation. Accordingly, all subsequent experiments were conducted using 2 μM UA to model a non-cytotoxic and physiologically relevant exposure condition.

Although UA increased mitochondrial biogenesis related-markers and respiratory capacity, no overt ultrastructural changes were detected by TEM. This apparent discrepancy between biochemical/functional adaptations and mitochondrial morphology is consistent with previous reports describing mitochondrial optimization without gross structural alterations detectable by conventional TEM (Ahsan et al., 2019). Notably, TEM samples in this study were prepared from centrifuged cell pellets rather than in situ-embedded adherent myotubes, a preparation that may disrupt myotube architecture, mix differentiated myotubes with residual myoblasts, and reduce the sensitivity to detect subtle network remodeling (Hinton et al., 2023). Together, these findings suggest that UA primarily promotes mitochondrial functional optimization rather than overt morphological remodeling or turnover during late-stage C2C12 differentiation.

In our C2C12 differentiation model (days 3–6), UA did not significantly alter mitophagy-related markers. However, a limitation of the present study is that autophagic/mitophagic flux was not directly assessed. Therefore, our data indicate only that UA did not change the steady-state levels of these markers under the tested conditions, rather than demonstrating the absence of mitophagy altogether. This observation agrees with reports that UA can activate autophagy without inducing mitophagy in ischemic neuronal models (Ahsan et al., 2019). By contrast, Ryu et al. (2016) showed that 5–10 μM UA applied for 12–24 h induces mitophagy in adult *C. elegans* and fully differentiated C2C12 myotubes (Ryu et al., 2016), while Andreux et al. (2019) reported a mitophagy-related molecular signature in human skeletal muscle after oral UA supplementation (Andreux et al., 2019). These differences likely reflect the distinct experimental context, since those studies used mature systems and either higher doses or shorter exposure times, whereas our model involved 2 μM UA for 72 h during an active differentiation phase (days 3–6). At this stage, mitochondrial biogenesis and oxidative adaptation may predominate over mitochondrial turnover (Sin et al., 2016) which could account for the lack of detectable mitophagy activation in our conditions.

We observed activation of the AMPKα–PGC-1α axis. AMPKα is a central energy sensor that can promote mitochondrial biogenesis through direct and indirect regulation of PGC-1α, and its activation has been linked to improved mitochondrial function in differentiating myotubes (Jäger et al., 2007). In human skeletal muscle, UA has been reported to increase PGC-1α and mitochondrial gene expression (Andreux et al., 2019), as well as respiratory chain components, including Complex II (Singh et al., 2022). In our model, however, UA did not significantly alter TOM20, OPA1 or MFN2, indicating that the observed functional effects were not accompanied by detectable changes in mitochondrial content or mitochondrial fusion markers. However, because direct measurements of mitochondrial biogenesis were not performed, our data should be interpreted as evidence of biogenesis-related signaling and mitochondrial functional adaptation rather than definitive proof of enhanced mitochondrial biogenesis. Notably, UA increased the abundance of OXPHOS Complex I and Complex II, which may help explain the higher maximal respiratory capacity observed after treatment, as both complexes represent major electron-entry sites into the respiratory chain and may support greater OXPHOS flux (Maekawa et al., 2020). Functionally, UA was associated with increased maximal respiratory capacity (FCCP) and spare respiratory capacity, consistent with improvements reported in skeletal muscle and cardiac models following UA treatment (50 μM for 24 h) (Andreux et al., 2019; Rodriguez et al., 2017). Importantly, basal respiration remained unchanged, suggesting that UA enhances mitochondrial reserve capacity rather than resting energy demand, an adaptation consistent with increased metabolic flexibility during muscle maturation. Although mitochondrial ROS production was not directly assessed in the present study, the dissociation between increased maximal respiration and unchanged basal respiration suggests improved mitochondrial efficiency rather than increased oxidative burden (Jones et al., 2021). Together, these findings suggest that UA enhances mitochondrial function during late-stage C2C12 differentiation without evidence of concomitant mitophagy activation or overt structural remodeling.

A key limitation of our study is that UA was administered only during days 3–6 of C2C12 differentiation, precluding evaluation of potential effects during earlier stages, when autophagy and mitophagy are more prominent. Moreover, we did not assess contractile function, fatigue resistance, or long-term myotube survival, so the impact of UA on muscle performance remains to be determined. These limitations highlight the need for future studies spanning the full myogenic program and incorporating functional outcomes in more physiologically relevant models. In this context, our findings help address an important gap in the literature by showing that UA can enhance mitochondrial respiratory capacity during late-stage C2C12 differentiation without detectable changes in steady-state mitophagy markers or mitochondrial ultrastructure. Collectively, these results suggest that UA may support mitochondrial oxidative adaptation during myogenic differentiation and provide a rationale for future *in vivo* studies.

Finally, our findings provide a mechanistic rationale to evaluate UA in vivo models of muscle regeneration (e.g., cardiotoxin injury, denervation, or hindlimb unloading), where the translational goal would be to determine whether UA-enhanced mitochondrial oxidative capacity in differentiating fibers translates to improved contractile recovery and functional outcomes. Moreover, our results help to expand current knowledge on the effects of urolithin A by identifying a previously unrecognized temporal window during differentiation in which it exerts its action, highlighting its relevance not only as a therapeutic approach but also as a potential preventive strategy in populations susceptible to muscle damage, such as athletes at risk of sports injuries and aging individuals.

## Data Availability

The raw data supporting the conclusions of this article will be made available by the authors, without undue reservation.
