# Supplementary figures and images for: Urolithin A enhances mitochondrial biogenesis-related markers and maximal respiratory capacity during C2C12 differentiation
Source: Front Cell Dev Biol. 2026 Jun 30;14:1854844. doi: 10.3389/fcell.2026.1854844 (PMC13364912; doi:10.3389/fcell.2026.1854844)

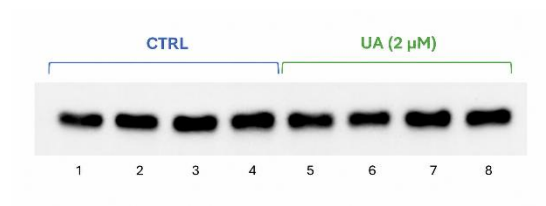

**MYH**

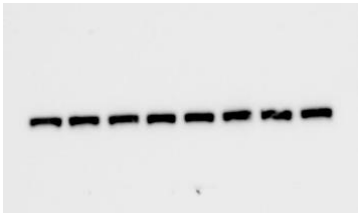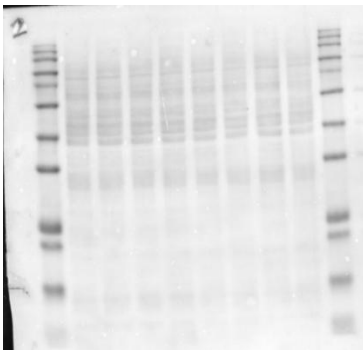

**Myogenin**

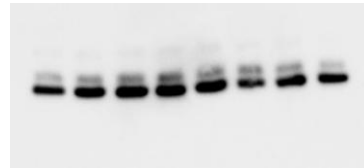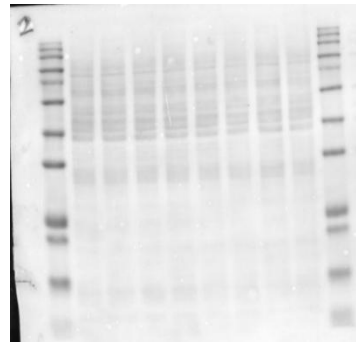

**MRF4**

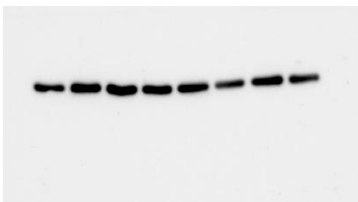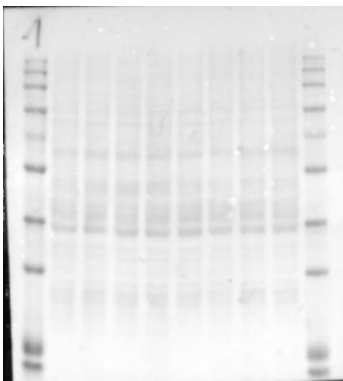

**MyoD**

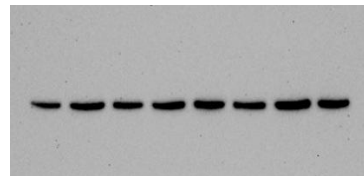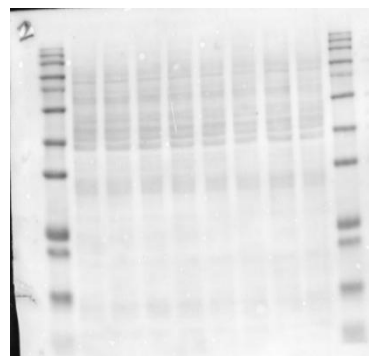

**p-ULK**

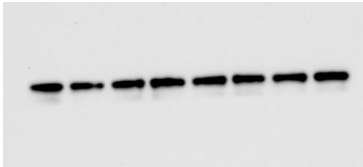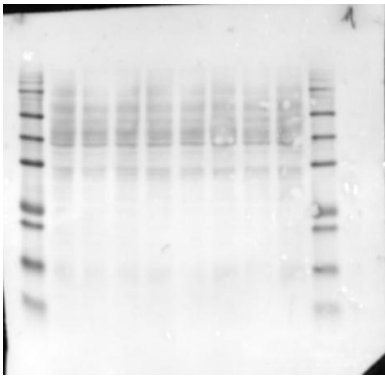

**BNIP3L**

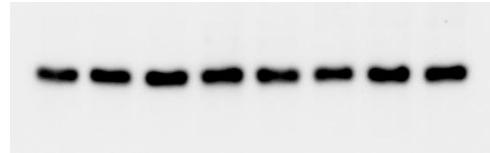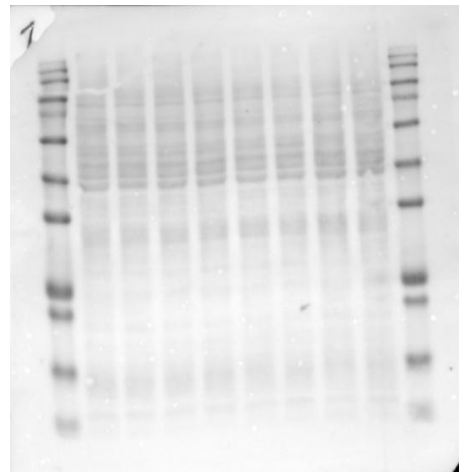

**P62**

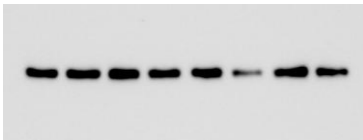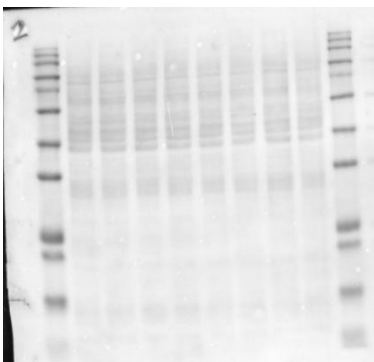

**LC3 I/II**

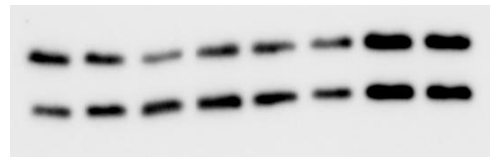

**p-AMPK**

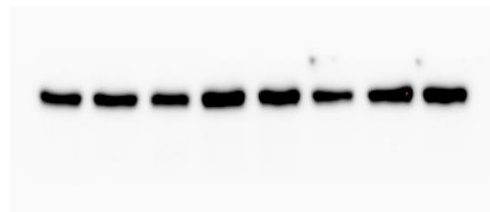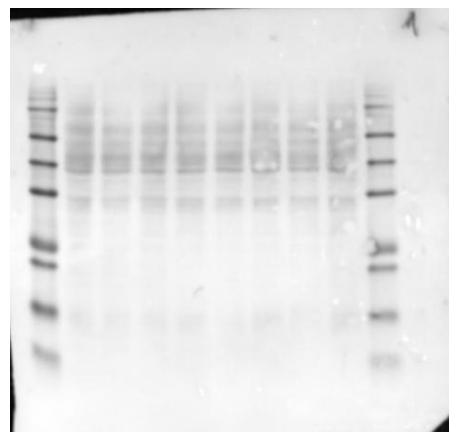

**PGC1 $\alpha$**

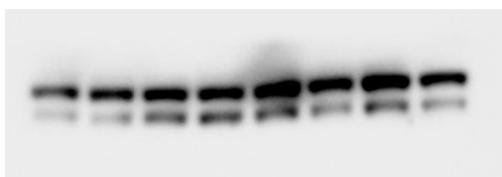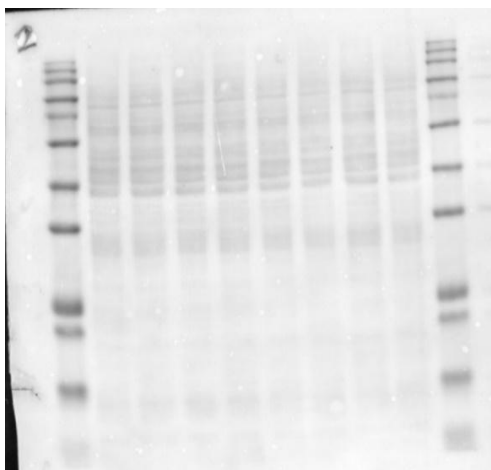

**MFN2**

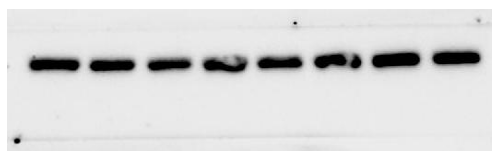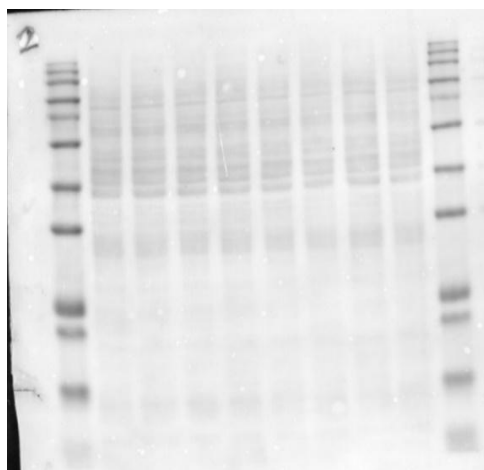

**TOM20**

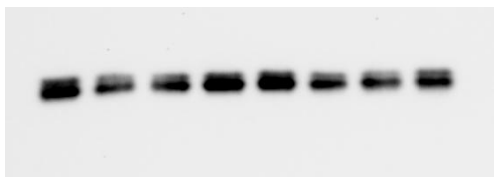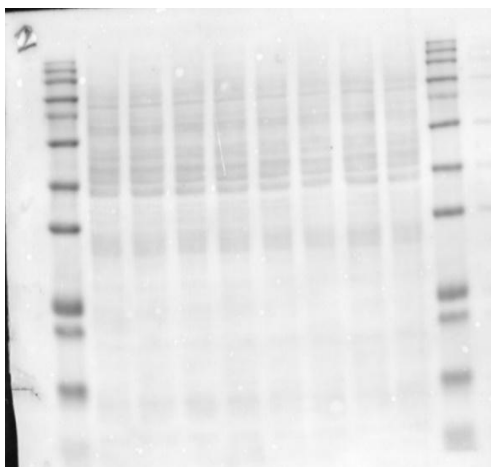

**OPA1**

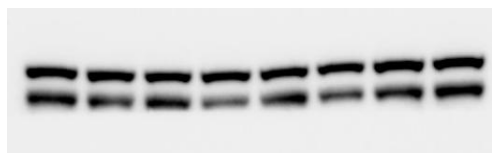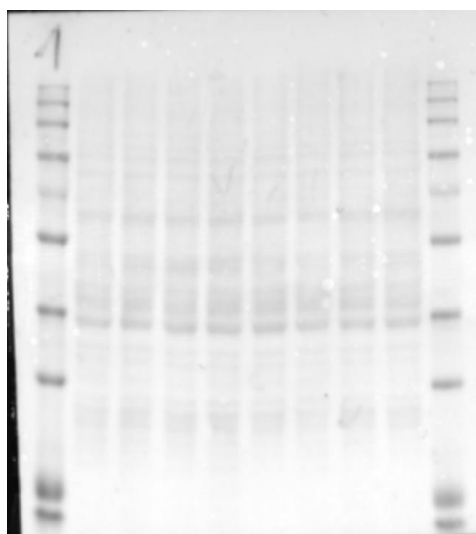

## OXPHOS

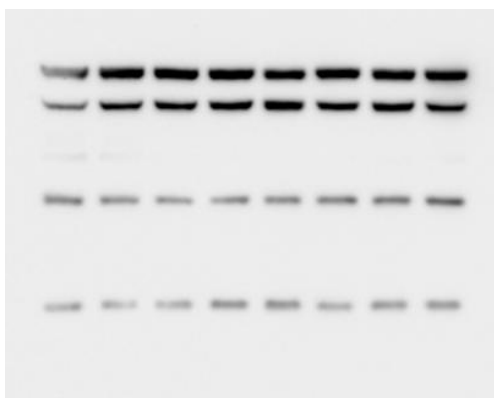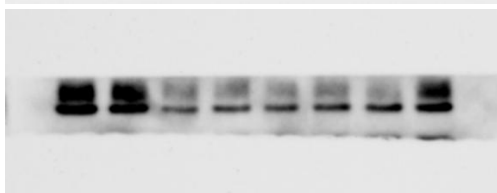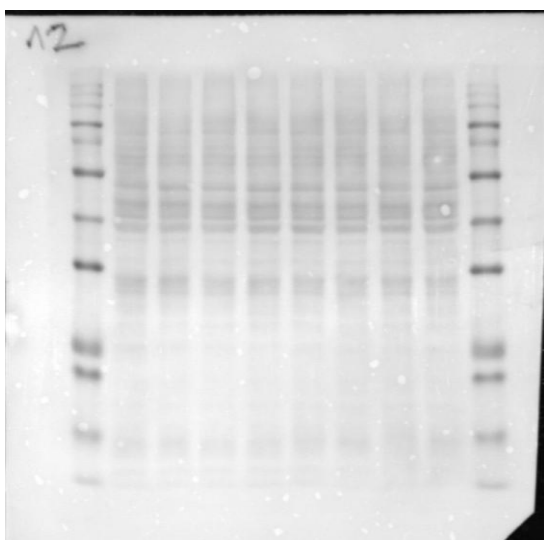

Supplement: Supplementary file 1 [file DataSheet1.pdf]
